# Evaluation of efficacy and safety of esmolol in treating patients with septic shock: A protocol for systematic review and meta-analysis

**DOI:** 10.1097/MD.0000000000029124

**Published:** 2022-04-01

**Authors:** Bin Hou, Ke Cai, Yue Li, Chunfang Hu, Xuehua Pu

**Affiliations:** Department of Critical Medicine, Taizhou People's Hospital, Taizhou, Jiangsu Province, China.

**Keywords:** efficacy, esmolol, meta, safety, septic shock

## Abstract

**Background:**

In septic shock cases, tachycardia and a hyperdynamic hemodynamic profile are characteristics of the condition. It has been reported that using beta antagonist esmolol constitutes a form of treatment to reduce heart rate to improve diastolic filling time and elevate cardiac output, which reduces vasopressor support. Still, there are controversial results. Therefore, in this study, the primary objective is to perform a meta-analysis by systematically evaluating the efficiency and security of using esmolol to treat septic shocks.

**Methods:**

A systematic literature search for relevant randomized controlled trials that report evaluations on the efficiency and safety of using esmolol to treat septic shock patients from their inception to February 2022 will be conducted in three databases containing publications in Chinese language (WanFang, Chinese BioMedical Literature Database, and China National Knowledge Infrastructure) and four databases containing English language publications (Cochrane Library, PubMed, Web of Science, and EMBASE). The screening of the relevant studies will be performed by a pair of authors independently, and the screening involves examining the title, abstract and full-text stages, data extraction, and bias risk assessment. The results are summarized through the fixed-effects and random-effects models, the respective models will be utilized for data pooling according to the heterogeneity of studies that will be included. Moreover, publication bias is assessed if more than ten studies are considered.

**Results:**

The results are a high-quality synthesis of the most recent evidence for esmolol usage in septic shock treatment.

**Conclusion:**

Up-to-date evidence will be provided through the results of this systematic review related to assessing the efficacy and safeness of esmolol usage in treating septic shock.

**Ethics and dissemination:**

Ethical permissions are not required as prepublished data are used.

**OSF registration number:**

DOI 10.17605/OSF.IO/SKEZ7

## 1 Introduction

Globally, septic shock is a predominant cause of mortality, often ranging between 30% to 50%. However, it may be even higher in certain locations.^[1,2]^ Admittedly, many advancements have been achieved in terms of treatment. However, vasopressors and fluid resuscitation remain the primary form of treatment for sepsis to maintain blood pressure and circulation to vital organs. However, such treatment measures can have fatal cardiac effects.^[3,4]^ Sepsis shocks primarily involve the heart. Almost half of all sepsis patients carry a poor prognosis of heart failure, and prolonged exposure to catecholamine remains a primary cause of sepsis-induced cardiac dysfunction, leading to sympathetic nerve overstimulation. Furthermore, studies have proven that patients with insufficient myocardial faces an elevated mortality rate compared to those without myocardial deficiency.^[5–7]^ Thus, strategies to avert extensive cardiomyocyte damage during the preliminary phase of sepsis are critically important for chances of surviving.

*β*-Adrenergic blockade can allow to control heart rate and minimize adversities owing to sympathetic overstimulation.^[8]^*β*-blockers can modulate the intrinsic response to *β*-adrenergic overstimulation to control the heart rate effectively.^[9]^ There are increasing reports on the beneficial effects of *β*-blockers in the treatment of septic shock and other severe terminal illnesses, which indicates an advantageous effect on mortality and morbidity.^[10–12]^ As an β1-adrenergic receptor blocker, esmolol has been tested by researchers in animal studies examining sepsis, and there have been promising outcomes in experimental models.^[13,14]^ Generally, esmolol is classified as a safe drug with a rapid onset of action. Moreover, it lasts for a short while and also protects the gut mucosa in sepsis.^[15]^ However, there are controversies in the efficacy and safety of using esmolol to treat septic shock. Thus, this meta-analysis assesses the efficacy and safety of using esmolol to treat septic shock. Additionally, it is a reference for clinicians.

## 2 Methods

The protocol of this study is registered in the Open Science Framework (register number: DOI 10.17605/OSF.IO/SKEZ7). The designing of the protocol adhered to the Cochrane Handbook for Systematic Reviews and the reporting follows the preferred reporting items for systematic review and meta-analysis protocol.

### 2.1 Inclusion criteria for study selection

#### 2.1.1 Type of studies

Each related randomized controlled trial (RCT) evaluating the usage of esmolol to treat septic shock shall be included, and there will be no language and publication constraints.

#### 2.1.2 Type of participants

Each participant diagnosed with septic shock or sepsis shall be included, and there are no constraints on sex, age, and race.

#### 2.1.3 Type of interventions

The experimental group should only be administered esmolol as an intervention. Meanwhile, the control's intervention can include different types of therapies, except any form of esmolol-based therapy.

#### 2.1.4 Type of outcome measures

Survival rate is the primary outcome. Meanwhile, mean arterial pressure, heart rate, central venous pressure, central venous oxygen saturation, and TnI are considered as secondary outcomes.

### 2.2 Search methods for the identification of studies

#### 2.2.1 Electronic searches

The authors will perform a comprehensive systematic literature search to identify related RCTs that assess the efficiency and safety of using esmolol for treating septic shock patients. The search will include all articles from inception till February 2022. Accordingly, in three databases containing publications in Chinese language (WanFang, Chinese BioMedical Literature Database, and China National Knowledge Infrastructure) and four databases containing English language publications (Cochrane Library, PubMed, Web of Science, and EMBASE). The search will include the following keywords: sepsis, septic shock, and esmolol.

#### 2.2.2 Searching other resources

The reference lists of all primary papers and related narrative review publications will be manually searched for additional references. Furthermore, the websites of the relevant manufacturers will also be searched for study information.

### 2.3 Data collection and analysis

#### 2.3.1 Selection of studies

The titles and abstracts of the papers will be separately screened by two authors. Subsequently, the articles will be coded as either “retrieve” (eligible or potentially eligible/unclear) or “do not retrieve.” Afterwards, the full-text study reports will be obtained of all possibly eligible studies. The pair of authors will then screen them independently for inclusion. We will also record all reasons for exclusion a study. All disagreements shall be mediated through discussions or by consulting a third author.

#### 2.3.2 Data extraction and management

A pair of authors will independently perform information extraction (e.g., first author, year of publication, baseline characteristics of patients, sample size, esmolol treatment, control) from the literature that satisfy the inclusion criteria. All disagreements shall be mediated through discussions or by consulting another independent author.

#### 2.3.3 Assessment of risk of bias in included studies

The authors will use the Cochrane risk of bias assessment tool to assess the bias risk in the articles.^[16]^ Accordingly, seven items shall be included: generation of random sequence, allocation concealment, blinding of participants and scholars, partial output data, selective reporting bias, and other bias. Accordingly, each item shall be categorized as either “Low Risk,” “Unclear,” or “High Risk.” Two authors will autonomously evaluate the methodological quality of the included trials. All disagreements shall be mediated via discussion or by consulting another author.

#### 2.3.4 Measures of treatment effect

In the case of continuous results, we will use the mean difference with 95% confidence intervals to present the extracted data. In the case of dichotomous results, we will use the risk ratio with 95% confidence intervals to compute extracted data.

#### 2.3.5 Assessment of heterogeneity

In this study, *I*^2^ statistic and Cochrane *Q* statistic tests are employed to assess heterogeneity. Heterogeneity assessment allows authors to gauge the feasibility to pool the data, and to perform meta-analysis. If *I*^2^ is higher than 50%, it will be considered as substantial heterogeneity, and subgroup analysis will be conducted to examine the potential factors from clinical or methodological heterogeneity.

#### 2.3.6 Assessment of reporting biases

If the number of included studies exceed 10, then we will assess the publication bias.

#### 2.3.7 Sensitivity analysis

For data that is eligible to be pooled, sensitivity analysis shall be employed to assess missing data, methodological quality, and the robustness of pooled results.

## 3 Discussion

The present protocol outlines a systemic methodology for a comprehensive review and meta-analysis to assess the effectiveness and level of safeness when using esmolol to treat septic shock. To the authors’ best knowledge, this will be the first attempt at analyzing the data from RCTs regarding this topic. Accordingly, the systemic review will be performed according to the established protocols and it is reported according to PRIMSA guidelines. It is our belief that this review will help treat septic shock more effectively. The main limitation is that the reliability of this review will be influenced by the quality of included trials.

## Author contributions

**Conceptualization:** Bin Hou, Chunfang Hu, Xuehua Pu.

**Data curation:** Bin Hou, Ke Cai, Yue Li, Chunfang Hu, Xuehua Pu.

**Formal analysis:** Bin Hou, Yue Li.

**Funding acquisition:** Chunfang Hu, Xuehua Pu.

**Investigation:** Bin Hou.

**Methodology:** Ke Cai, Yue Li, Xuehua Pu.

**Project administration:** Bin Hou, Yue Li.

**Resources:** Xuehua Pu.

**Software:** Bin Hou, Ke Cai, Chunfang Hu.

**Supervision:** Yue Li, Xuehua Pu.

**Validation:** Bin Hou, Ke Cai.

**Visualization:** Bin Hou, Xuehua Pu.

**Writing – original draft:** Bin Hou, Xuehua Pu.

**Writing – review & editing:** Xuehua Pu.
